# Management and outcome of patients with chronic myeloid leukemia in blast phase in the tyrosine kinase inhibitor era – analysis of the European LeukemiaNet Blast Phase Registry

**DOI:** 10.1038/s41375-024-02204-y

**Published:** 2024-03-28

**Authors:** Annamaria Brioli, Elza Lomaia, Christian Fabisch, Tomasz Sacha, Hana Klamova, Elena Morozova, Aleksandra Golos, Philipp Ernst, Ulla Olsson-Stromberg, Daniela Zackova, Franck E. Nicolini, Han Bao, Fausto Castagnetti, Elzbieta Patkowska, Jiri Mayer, Klaus Hirschbühl, Helena Podgornik, Edyta Paczkowska, Anne Parry, Thomas Ernst, Astghik Voskanyan, Elzbieta Szczepanek, Susanne Saussele, Georg-Nikolaus Franke, Alexander Kiani, Edgar Faber, Stefan Krause, Luis Felipe Casado, Krzysztof Lewandowski, Matthias Eder, Peter Anhut, Justyna Gil, Thomas Südhoff, Holger Hebart, Sonja Heibl, Markus Pfirrmann, Andreas Hochhaus, Michael Lauseker

**Affiliations:** 1 Klinik und Poliklinik für Innere Medizin C, Hämatologie und Onkologie, Universitätsmedizin Greifswald, Greifswald, Germany; 2 Klinik für Innere Medizin II, Universitätsklinikum Jena, Comprehensive Cancer Center Central Germany, Campus Jena, Jena, Germany; 3 Klinik für Hämatologie, Hämostaseologie, Onkologie und Stammzelltransplantation, Medizinische Hochschule Hannover, Hannover, Germany; 4 Research Department of Immuno-Oncology, Almazov National Medical Research Centre, Saint Petersburg, Russian Federation; 5 Department of Hematology, Jagiellonian University Medical College, Krakow, Poland; 6 Institute of Hematology and Blood Transfusion, Prague, Czech Republic; 7 Raisa Gorbacheva memorial Research Institute for Pediatric Oncology, Hematology, Transplantation, First State Pavlov Medical University of Saint Petersburg, Saint Petersburg, Russian Federation; 8 Hematooncology Department, Copernicus Memorial Hospital, Lodz, Poland; 9 Department of Hematology, University Hospital Uppsala, Uppsala, Sweden; 10 Department of Internal Medicine, Hematology and Oncology, University Hospital Brno and Masaryk University, Brno, Czech Republic; 11 Centre Léon Bérard, Hématology Départment and CRCL INSERM U590, Lyon, France; 12 Institut für Medizinische Informationsverarbeitung, Biometrie und Epidemiologie (IBE), Medizinische Fakultät, Ludwig-Maximilians-Universität München, Munich, Germany; 13 IRCCS Azienda Ospedaliero-Universitaria di Bologna, Istituto di Ematologia “Seràgnoli”, Bologna, Italy; 14 Dipartimento di Medicina Specialistica, Diagnostica e Sperimentale, Università di Bologna, Bologna, Italy; 15 Hematology Department, Institute of Hematology and Transfusion Medicine, Warsaw, Poland; 16 Hematology and Oncology, Faculty of Medicine, University of Augsburg, Augsburg, Germany; 17 Department of Haematology, University Medical Centre Ljubljana, Ljubljana, Slovenia; 18 Faculty of Pharmacy, University of Ljubljana, Ljubljana, Slovenia; 19 Department of General Pathology, Pomeranian Medical University in Szczecin, Szczecin, Poland; 20 Centre Hospitalier Annecy Genevois, Annecy, France; 21 Hematology Center after Prof. R. Yeolyan, Yerevan, Armenia; 22 Department of Hematology, Jagiellonian University Medical College, Cracow, Poland; 23 III. Med. Klinik, Med. Fakultät Mannheim, Universität Heidelberg, Mannheim, Germany; 24 University of Leipzig Medical Center, Department of Hematology, Cellular Therapy, Hemostaseology and Infectious Diseases, Comprehensive Cancer Center Central Germany, Campus Leipzig, Leipzig, Germany; 25 Medizinische Klinik IV, Klinikum Bayreuth GmbH, Bayreuth, and Comprehensive Cancer Center Erlangen-EMN, Bayreuth, Germany; 26 Department of Hemato-Oncology, University Hospital Olomouc, Faculty of Medicine and Dentistry, Palacky University in Olomouc, Olomouc, Czech Republic; 27 Uniklinik Erlangen, Medizinische Klinik 5, Erlangen, Germany; 28 Servicio de Hematología, Hospital General Universitario de Toledo, Toledo, Spain; 29 Department of Hematology & Bone Marrow Transplantation, Poznań University of Medical Sciences, Poznań, Poland; 30 Onkologische Schwerpunktpraxis Anhut, Kronach, Germany; 31 Oncology Centre of the Podkarpackie Province, Department of Hematooncology, Brzozow, Poland; 32 Klinikum Passau, Klinik für Onkologie, Hämatologie und Palliativmedizin, Passau, Germany; 33 Zentrum für Innere Medizin, Hämatologie/Onkologie, Stauferklinikum Schwäbisch Gmünd, Mutlangen, Germany; 34 Abteilung für Innere Medizin IV, Klinikum Wels-Grieskirchen, Wels, Austria

**Keywords:** Chronic myeloid leukaemia, Epidemiology

## Abstract

Blast phase (BP) of chronic myeloid leukemia (CML) still represents an unmet clinical need with a dismal prognosis. Due to the rarity of the condition and the heterogeneity of the biology and clinical presentation, prospective trials and concise treatment recommendations are lacking. Here we present the analysis of the European LeukemiaNet Blast Phase Registry, an international collection of the clinical presentation, treatment and outcome of blast phases which had been diagnosed in CML patients after 2015. Data reveal the expected heterogeneity of the entity, lacking a clear treatment standard. Outcomes remain dismal, with a median overall survival of 23.8 months (median follow up 27.8 months). Allogeneic stem cell transplantation (alloSCT) increases the rate of deep molecular responses. De novo BP and BP evolving from a previous CML do show slightly different features, suggesting a different biology between the two entities. Data show that outside clinical trials and in a real-world setting treatment of blast phase is individualized according to disease- and patient-related characteristics, with the aim of blast clearance prior to allogeneic stem cell transplantation. AlloSCT should be offered to all patients eligible for this procedure.

## Introduction

Since the advent of tyrosine kinase inhibitors (TKI), chronic myeloid leukemia (CML) has become a paradigm of successful targeted treatment for hematologic malignancies [1]. Inhibition of the abnormal tyrosine kinase BCR::ABL1 has transformed this once deadly disease in a controllable entity, with most patients having a normal life expectancy [2] and with some patients being able to successfully stop treatment after having achieved a deep molecular response [3–9]. Despite the success of CML treatment, progression to blast phase (BP), an aggressive form of acute leukemia associated with unfavorable prognosis, may occur [10–12]. Prior to the introduction of TKI virtually all patients who did not undergo an allogeneic stem cell transplantation (alloSCT) progressed to BP, with a mortality rate of more than 20% per year in the majority of studies [13, 14]. The implementation of TKI into CML management has dramatically changed this scenario, and nowadays less than 5% of patients develop BP disease [10]. Nevertheless, in contrast to major advances in chronic phase (CP) CML, outcome is still dismal for this important minority of patients [15, 16]. BP can be of myeloid, lymphoid, mixed, or megakaryoblastic phenotype, can evolve from a previous CML, but can also be the disease presentation at diagnosis, the so-called de novo BP [11, 12]. Treatment of BP with TKI monotherapy often leads to short-term remissions, with inevitably almost all patients experiencing a disease relapse if alloSCT is not performed [17–22]. Due to the rarity of this condition and the lacking of clinical trials most of the available recommendations are based on retrospective analyses and expert consensus [12, 23]. Further, the definition of BP is not homogeneous. The ELN recommendation panel considers a BP when the proportion of blasts in peripheral blood and/or bone marrow is ≥30% [24]. Conversely, the WHO define a BP already with a percentage of blasts ≥20% [25]. Although the prognosis of patients with 20–29% blasts seems to be similar to the one of patients with ≥30% of blasts [26], definitions should be harmonized, in order to have uniform inclusion criteria into clinical trials and registries. The biology of BP also poses an additional challenge. Although some features of BP are well characterized [27, 28], it remains unclear how the biology of the disease might have changed after the introduction of TKI. To address these open issues, and to set the basis for the set-up of future clinical trials, an international registry was established with the aim of collecting biological and clinical data on CML in BP to better understand disease characteristics and treatment approaches in the TKI era.

## Materials and methods

The European LeukemiaNet Blast Phase Registry is an ongoing international project collecting data of patients diagnosed with CML-BP. Participating countries are Germany, Poland, Russia, Czech Republic, Armenia, France, Italy, Austria, Sweden, Slovenia, and Spain. Patients can be included if they have a BP evolving from a previous CML or present with a de novo BP. Patients must be older than 18 years of age at the time of BP development. To obtain a realistic picture of CML-BP in recent years, only patients with a BP diagnosed after January 1st, 2015 can be recruited. This allowed for a wider inclusion of patients treated with second-generation TKIs, although it should be noted that the availability of TKIs varied between participating countries (Supplementary Table 1). The project was approved by the respective ethic committee in each center and was conducted in accordance with the principle of good clinical practice and the declaration of Helsinki. All living patients included in the registry provided written informed consent. For patients who were already deceased at the time of the enrollment, informed consent was deemed not necessary by the ethic committee. In these cases, fewer personal data were collected to protect patients’ confidentiality.

### Statistical analysis

Metrical covariates were compared using the Mann–Whitney–Wilcoxon test. For categorical covariates, Fisher’s exact test was used. Survival was analyzed using Kaplan–Meier curves and the log-rank test. *P* < 0.05 were considered significant. Due to the exploratory character of this work, no *p*-value adjustment was applied; thus, all *p*-values have to be interpreted descriptively. Responses were evaluated at predetermined time points +/−1 month.As an example, for the 3-month evaluation, all responses recorded between month 2 and month 4 were evaluated. If multiple responses were present in this time frame, the most proximate analysis was chosen. Patients deceased prior the evaluation time point are reported as such. All analyses were performed with SAS 9.4 or R 4.0.2.

## Results

**Fig. 1:**
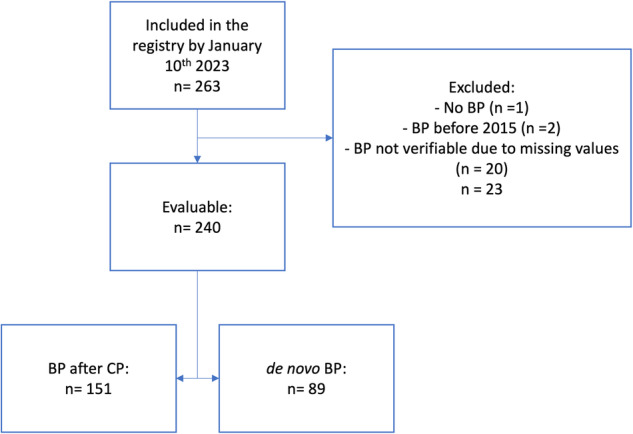
Consort diagram of patients included in the registry and in the analysis. BP blast phase, CP chronic phase.

At the cut-off date of January 10, 2023, 263 patients have been documented in the registry and 240 formed the core of the present analysis. Twenty-three patients were excluded due to lack of accurate diagnosis of BP, evidence for BP before January 1st, 2015, or incomplete data. A flow chart of patients documented and analyzed is presented in Fig. 1.

Patients’ characteristics are reported in Table 1. The availability of TKI by participating countries is detailed in Supplementary Table 1. Median age at diagnosis of BP was 49 years and 60.0% of patients were male. Eighty-nine patients (37.0%) where diagnosed with a de novo BP. For those patients diagnosed with a previous CML, median time between CML diagnosis and evolution to BP was 29.1 months (range, 1–378). Two BP occurred in patients that had stopped TKI within a treatment free remission concept.

**Table 1:** Patients and disease characteristics.

Variable		*N*	
**Patient-related**			
Sex, male, *n* (%)		240	144 (60.0%)
Age at CML diagnosis (yrs), median (range)		240	45 (13–86)
Age at onset of CML-BP (yrs), median (range)		240	49 (18–86)
Phase at diagnosis, *n* (%)	CML-CP	240	151 (62.9%)
	CML-BP		89 (37.0%)
Onset of CML-BP after diagnosis of CML-CP (mo), median (range)		151	29.1 (0.1–378)
ELTS Score at diagnosis of CML-CP, *n* (%)	Low risk, *n* (%)	151	36 (39.6%)
	Intermediate risk, *n* (%)		33 (36.3%)
	High risk, *n* (%)		22 (24.2%)
**CML-related**			
Morphology of CML-BP	Myeloid	233	117 (50.2%)
	Lymphoid		71 (30.5%)
	Mixed		10 (4.3%)
	Megakaryoblastic		3 (1.3%)
	Unknown		32 (13.7%)
	Not reported	7	
Additional chromosomal abnormalities (ACAs) at onset of CML-BP, yes, *n* (%)	Complex karyotype	174	52 (29.9%)
	Chr. 3q26.2 rearrangements		9 (5.2%)
	−7/−7q		12 (6.9%)
	+8		23 (13.2%)
	+Ph		20 (11.5%)
	Others		50 (28.7%)
	Not reported	66	
High risk ACAs^a^, yes, *n* (%)		174	72 (41.4%)
	Not reported	66	
CNS involvement, yes, *n* (%)		214	21 (9.8%)
	Not reported	26	
Extramedullary disease, yes, *n* (%)		220	43 (19.5%)
	Not reported	20	

*M* male, *F* female, *CML* chronic myeloid leukemia, *yrs* years, *CML-BP* chronic myeloid leukemia blast phase, *CML-CP* chronic myeloid leukemia chronic phase, *mo* months, *ELTS* EUTOS long term survival, *chr.* chromosome, *CNS* central nervous system.

^a^High risk ACAs: +8, +Ph, i[17q], +17, +19, +21, 11q23 and 3q26.2 rearrangements, −7/−7q abnormalities, complex karyotype.

Information about additional chromosomal abnormalities (ACAs) was available in 174/240 patients, of which 101 (58.0%) had at least one ACA. The most frequent high-risk abnormality (29.9%) was a complex karyotype, followed by +8, additional Philadelphia chromosome (+22q-), deletion of chromosome 7/7q, and abnormalities of chromosome 3q26.2 (Table 1).

*BCR::ABL1* transcript type was e13a2 and/or e14a2 in 90.7% (117/129) of patients. Twelve patients had atypical transcripts (Table 2 and Supplementary Table 2). *BCR::ABL1* mutations occurred in 28.0% of the patients. As expected, the most frequently *BCR::ABL1* mutation reported was T315I (12 cases), followed by E255K (*n* = 11), F317L (*n* = 5), and Y253H (*n* = 5) (Table 2). Eleven patients had more than one *BCR::ABL1* mutation. Thirty mutations in genes other than *BCR::ABL1* were reported in 21 patients investigated (Supplementary Table 3). The type of mutation was heterogeneous, with no unifying pattern.

**Table 2:** Type of *BCR::ABL1* transcript and occurrence of mutations for all patients and for patients with secondary or de novo CML-BP.

*BCR::ABL1* transcript type (*N* = 129)	All patients *n*	Secondary CML-BP *n*	de novo CML-BP *n*
e13a2	47	28	19
e14a2	47	31	16
e13a2 + e14a2	23	14	9
e1a2	7	2	5
e6a2	1	0	1
e8a2	1	1	0
e19a2	2	0	2
e13a3	1	0	1
Not reported	111	75	36
Presence of *BCR::ABL1* mutations (*N* = 166)			
Yes	46 (27.7%)	39 (37.5%)	7 (11.3%)
Not reported	74	47	27
*BCR::ABL1* mutations (*N* = 59)^a^			
T315I	12	9	3
E255K	11	10	1
F317L	5	5	0
Y253H	5	5	0
F359V	3	3	0
G250E	2	2	0
G250R	2	1	1
V299L	2	2	0
E459K	1	1	0
F317I	2	2	0
H396R	2	2	0
Q252H	1	1	0
Y393C	1	1	0
V280I	1	1	0
Others	7 ( + 2 unknown)	5 ( + 2 unknown)	2
Mutations/alterations in genes outside *BCR::ABL1* (*N* = 30)^b^			
WT1	5	4	1
RUNX1^c^	1^c^ + 3	1^c^ + 2	1
ASXL1^c^	1^c^ + 1	1^c^ + 1	0
FLT3-ITD^c^	1	1	0
FLT3-TKD	1	1	0
BCORL1^d^	1	1	0
IDH1^d^	1	1	0
KRAS^c^	1	1	0
NRAS^e^	1^e^	1^e^	0
CBFB:MYH11	1	1	0
MECOM	1	1	0
JAK2^f^	1	1	0
NPM1	1	0	1
TP53^e,^^g^	1 + 1^e^ + 1^g^	1 + 1^e^ + 1^g^	0
ZRSR2^e^	1	1	0
EZH2^e^	1	1	0
STAG2^g^	1	1	0
GATA2	1	0	1
NOTCH1^f^	1	1	0
EV1 overexpression	1	1	0
Not reported	210	125	85

*CML-BP* chronic myeloid leukemia blast phase.

^a^11 patients had more than one *BCR::ABL1* mutation.

^b^5 patients had more than one mutation.

^c^same patient.

^d^same patient.

^e^same patient.

^f^same patient.

^g^same patient.

Information regarding central nervous system (CNS) and extramedullary involvement were reported in 214 and 220 patients respectively. Of these 9.8% had CNS involvement and 19.5% extramedullary manifestations.

ELTS score at the time of CML diagnosis was available for all 151 patients with a previous CML, and was low risk in 39.6%, intermediate risk in 36.3% and high-risk in 24.2% of them (Table 1).

### Treatment

Treatment of BP was heterogeneous with regard to sites and individual patient. The median number of lines of therapy for BP was 3. One-hundred and thirty-two out of 240 patients (55.0%) received at least one alloSCT for their BP during the course of the disease. Additional 16 patients had previously received an alloSCT in the CML-CP and progressed to BP after transplant. Regarding treatment given at the first occurrence of BP, 232 patients had complete data and could be analyzed. TKIs were the cornerstone of BP treatment, with only 16.9% of patients not receiving a TKI. The combination employed most frequently was a TKI plus chemotherapy (42.7% of cases), followed by TKI alone (21.1%). TKI plus chemotherapy and alloSCT was used in 15.1%, whilst 10 patients received TKI and alloSCT. For those patients receiving alloSCT, the median time from BP diagnosis to transplantation was 6 months.

At initial therapy, dasatinib was the most frequently used TKI (32.3%), followed by imatinib and ponatinib (26.7% and 13.4%, respectively). Median duration of TKI therapy was 121 days for dasatinib, 94 days for imatinib and nilotinib and 87 days for ponatinib. Bosutinib was given in first line in 4 patients only, with a median duration of therapy of 187 days. The different treatment combinations in first line and for all lines of treatment as well as the frequency of the usage of the various TKIs is reported in Supplementary Tables 3 and 4.

CNS prophylaxis or CNS therapy was used in 44 patients. Twenty-three additional patients received systemic chemotherapy containing drugs active on the CNS, such as methotrexate and lomustine.

Looking at factors that might influence choice of first line treatment, patients treated with dasatinib were more likely to be younger than patients receiving other TKIs (median: 45 vs. 50 years, *p* = 0.0291). Similarly, patients receiving alloSCT were in median younger than patients not being transplanted (median 43 vs. 50 years, *p* = 0.010). In contrast, ponatinib patients were older (median 53 vs 47 years, *p* = 0.0219). Patients with a shorter CP duration before evolution in BP were more likely to be treated with dasatinib (median 16 vs. 34 months, *p* = 0.0060) and alloSCT (median 14 vs. 31 months, *p* = 0.0394). Imatinib was given predominantly in cases where no *BCR::ABL1* mutations were present with a frequency of 29.7% compared to only 9.5% in the presence of a *BCR::ABL1* mutation (*p* = 0.0111). Ponatinib was the more frequently used TKI when *BCR::ABL1* mutations were present (35.7% vs. 11.0%, *p* = 0.0007). Treatment was influenced also by the phenotype of BP. Chemotherapy and alloSCT were used more often in patients with a lymphoid BP (LyBP) as compared with patients with a myeloid BP (MyBP) (odds ratio [OR] 5.10, 95%-confidence interval [CI]: 2.1–12.1, *p* < 0.0001 and OR 2.37, 95%-CI: 1.2–4.8, *p* = 0.0181, respectively).

### Responses

Responses were evaluated at three and at 6 months after onset of BP. Three months response data were evaluable for 134 patients, including 21 (15.7%) patients with early death during the first 3 months after diagnosis of BP. Thirty-four patients (25.4%) did not achieve any response and remained in BP. Sixty-seven (50.0%) and 38 (28.4%) patients achieved at least a complete hematologic response (CHR) and a complete cytogenetic response (CCyR), respectively. Major molecular response (MMR) was achieved in 27 patients (20.1%), with 12 (9.0%) each achieving MR^4.5^ (*BCR::ABL1* transcript levels ≤0.0032% on the international scale, IS [29]) and MR^5^ (*BCR::ABL1* transcript levels ≤0.001% IS [29]) (Table 3). When censoring for alloSCT, results did not change significantly, since only three patients had an outcome measurement after alloSCT.

**Table 3:** Responses to therapy of CML-BP.

	Responses at 3 months including alloSCT (*N* = 134)	Responses at 6 months including alloSCT (*N* = 112)	Best responses censored alloSCT (*N* = 123)	Best responses including alloSCT (*N* = 184)
No response	34	19	37	47
Return to CML-CP	12	3	5	5
CHR + PCyR	29	9	18	11
CCyR or BCR::ABL1 <1%	11	4	16	12
MMR	11	14	15	9
MR^4^	4	3	10	11
MR^4.5^	6	6	7	12
MR^5^	6	21	15	77
Deceased	21	33		
Observation less than 3 resp. 6 months	20	24		
Not reported	86	104		

MMR: *BCR::ABL1* ≤ 0.1% international scale (IS); MR^4^: *BCR::ABL1* ≤ 0.01% IS; MR^4.5^: *BCR::ABL1* ≤ 0.0032% IS; MR^5^: *BCR::ABL1* ≤ 0.001% IS.

*CML-BP* chronic myeloid leukemia blast phase, *alloSCT* allogeneic stem cell transplantation, *CML-CP* chronic myeloid leukemia chronic phase, *CHR* complete hematologic response, *CCyR* complete cytogenetic response, *PCyR* partial cytogenetic response (Ph + ≤35%), *MMR* major molecular remission.

Six-month data were evaluable for 112 patients, of which 33 (29.5%) had died before this milestone. Of evaluable patients, 57 (50.9%) achieved at least a CHR and 48 (42.9) achieved a CCyR at 6 months. MMR was achieved in 44 patients (39.3%), MR^4.5^ and MR^5^ in 27 (24.1%) and 21 (18.8%), respectively. The proportion of patients who did not respond to treatment was 17.0% (19 patients). Eleven of these results were obtained after alloSCT. Best response achieved at any time during treatment is available in 184 patients (Table 3). Best response results depend heavily on individual observation times, as patients with shorter observation times might still improve their responses in the future. For this reason, these results are to be interpreted purely descriptive, and, in contrast to the 3- and 6-months’ time-points, should not be generalized. When censoring responses for alloSCT, the rates of CHR, CCyR, MMR and MR^5^ was 68.9%, 51.2%, 38.2%, and 12.2%, respectively. After alloSCT, the proportion of patients achieving an MMR increased to 59.2%, with 41.8% of patients achieving MR^5^.

### Comparison between BP from previously diagnosed CML and de novo BP

Given the relatively high number of patients with de novo BP in our series, differences between these patients and those whose BP evolved from a preceding CP (secondary BP) were explored. Age distribution was similar in the two groups (Table 4). The proportion of female patients was higher in de novo BP (47.2% vs. 35.8%), although the difference was not statistically significant. The phenotypic presentation between de novo BP and secondary BP was comparable, yet atypical transcripts were more frequent in de novo BP (3.9% vs. 17.0%, *p* = 0.028). High risk ACAs and mutations in the *BCR::ABL1* gene were more frequent in secondary BP patients. Interestingly there was no significant difference between the two groups regarding the proportion of patients presenting with CNS or extramedullary involvement (Table 4).

**Table 4:** Comparison between CML-BP as evolution of a chronic phase (secondary BP) and de novo CML-BP.

Variable		Secondary CML-BP (*N* = 151)	De novo CML-BP (*N* = 89)
Patient-related			
Sex, male, *n* (%)		97 (64.2%)	47 (52.8%)
Age at onset of CML-BP (yrs), median (range)		49 (18–85)	48 (20–86)
CML-related			
Morphology of CML-BP, n/N (%)	Myeloid	75/148 (50.7%)	42/85 (49.4%)
	Lymphoid	46/148 (31.1%)	25/85 (29.4%)
	Mixed	5/148 (3.4%)	5/85 (5.9%)
	Megakaryoblastic	1/148 (0.7%)	2/85 (2.4%)
	Unknown	21/148 (14.2%)	11/85 (12.9%)
	Not reported	3 (2.0%)	4 (4.5%)
Additional chromosomal abnormalities (ACAs), yes, n/N(%)	Complex karyotype	38/101 (37.6%)	14/73 (19.2%)
	Chr. 3q26.2 rearrangements	6/101 (5.9%)	3/73 (4.1%)
	−7/−7q	12/101 (11.9%)	0/73 (0%)
	+8	23/101 (22.8%)	0/73 (0%)
	others	41/101 (40.6%)	22/73 (30.1%)
	Not reported	50 (33.1%)	16 (18.0%)
High risk ACAs^a^, yes, n/N(%)		56/101 (55.4%)	16/73 (21.9%)
	Not reported	50 (33.1%)	16 (18.0%)
Mutations in *BCR::ABL1*, yes, n/N(%)		39/104 (37.5%)	7/62 (11.3%)
	Not reported	47 (31.1%)	27 (30.3%)
CNS involvement, yes, n/N(%)		10/133 (7.5%)	11/81 (13.6%)
	Not reported	18 (11.9%)	8 (9.0%)
Extramedullary disease, yes, n/N(%)		27/136 (19.9%)	16/84 (19.0%)
	Not reported	15 (9.9%)	5 (5.6%)

*CML-BP* chronic myeloid leukemia blast phase, *M* male, *F* female, *yrs* years, *chr.* chromosome, *CNS* central nervous system.

^a^High risk ACAs: +8, +Ph, i[17q], +17, +19, +21, 11q23 and 3q26.2 rearrangements, −7/7q abnormalities, complex karyotype.

Patients diagnosed with de novo BP received most frequently imatinib (OR 13.09, 95%-CI: [6.44; 26.63], *p* < 0.001) as first line treatment. Only patients with a secondary BP received ponatinib in first line (21.4%, *p* < 0.0001). The other treatments were relatively balanced between the two groups (Supplementary Table 3).

Regarding treatment efficacy, response distributions at 3 and 6 months were not significantly different when comparing secondary BP to de novo BP. Nevertheless, patients with de novo BP tended to have a better outcome at the 6-month time-point, although significance was not reached (Mann–Whitney test *p* = 0.06)

### Survival

**Fig. 2:**
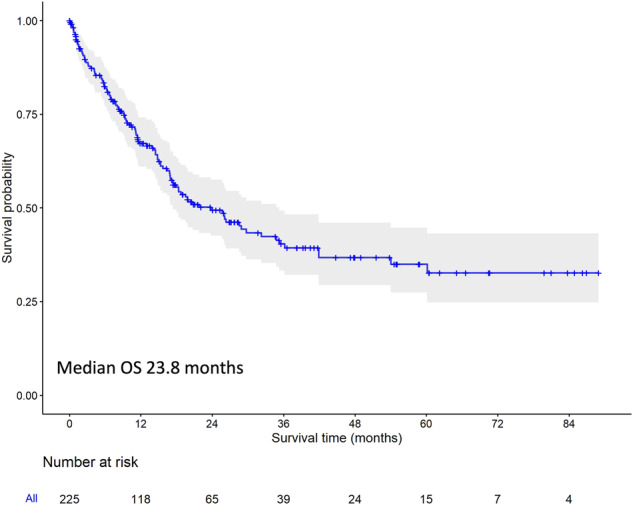
Overall survival from diagnosis of blast phase (all population). Median follow up 27.8 months.

**Fig. 3:**
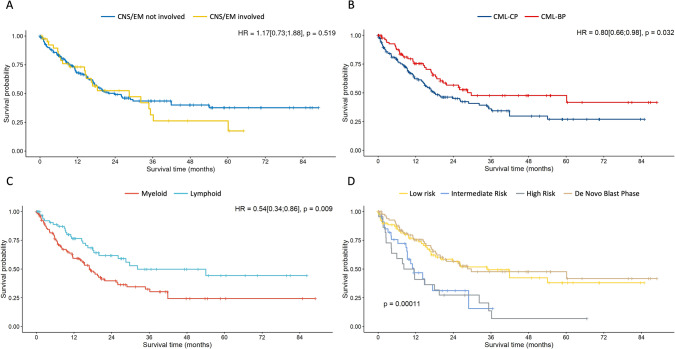
Overall survival from diagnosis of blast phase according to different subgroups. **A** overall survival for patient with central nervous system (CNS) and/or extramedullary (EM) involvement compared to patients without CNS and extramedullary involvement; **B** Overall survival for patient with de novo blast phase (CML-BP) and blast phase evolving from a previous chronic phase (CML-CP); **C** overall survival according to disease phenotype (myeloid vs. lymphoid); **D** overall survival according to disease phase (de novo) and ELTS score at the time of chronic phase (ELTS low, ELTS intermediate, ELTS high) [low ELTS vs. de novo HR 1.09 [95% CI: 0.68–1.75], *p* = 0.713, low ELTS vs. intermediate ELTS HR 2.35 [95% CI 1.35–4.11], *p* = 0.003, low ELTS vs. high ELTS HR 2.75 [95% CI: 1.57–4.81], *p* < 0.001]. All *p* values have to be interpreted as exploratory.

With a median follow up of 27.8 months, median overall survival was 23.8 months (95% CI: 17.0–34.8, Fig. 2). There was almost no difference in survival in patients with CNS or extramedullary involvement as compared to patients without these high-risk characteristics (median 28.5 vs. 23.8 months, HR 1.17 [95% CI: 0.73–1.88], *p* = 0.519, Fig. 3A). Conversely, patients with de novo BP had a better outcome then patients with a preceding CP (median 29.7 vs. 18.0 months, HR 0.80 [95% CI: 0.66–0.98], *p* = 0.032, Fig. 3B), as did patients with a lymphoid phenotype (median 32.2 vs. 17.0 months for LyBP vs. MyBP, HR 0.54 [95% CI: 0.34–0.86], *p* = 0.009, Fig. 3C). Patients with a low ELTS score at diagnosis of CML [30] and patients with de novo BP had rather comparable outcomes (median 34.8 vs. 29.7 months for low ELTS and de novo BP respectively, HR 1.09 [95% CI: 0.68–1.75], *p* = 0.713), significantly better than the outcome of patients with intermediate (11.4 months, HR 2.35 [95% CI: 1.35–4.11], *p* = 0.003) or high (9.9 months, HR 2.75 [95% CI: 1.57–4.81], *p* < 0.001) ELTS score (Fig. 3D). Patients with ≥30% blasts had a 1.7-times higher hazard of dying than patients with 20–29% blasts, however, the differences were not significant (median survival: 20.7 for patients with 20–29% blasts vs. 15.7 months for patients with ≥30% blasts). When differentiating between de novo BP and secondary BP, survival was comparable between both blast categories in de novo patients (HR: 1.19 [95% CI: 0.47–2.99], *p* = 0.706). Conversely, a minor and not significant difference between patients with ≥30% and 20–29% blasts (HR: 2.10 [95% CI: 0.94–4.74], *p* = 0.072) was found in patients with preceding CP.

## Discussion

Optimization of the management of CML in BP is still an unmet clinical need. Recently, an international consortium of experts on pediatric CML published treatment recommendations for the management of pediatric CML in BP [23]. The effort of this consortium is the first attempt to provide recommendations for a consistent therapy in CML-BP. A similar consensus for adult patients is still lacking.

Importantly, in the adult population, even when large retrospective analyses are available, these are mostly monocentric [31, 32], and thus accounting only partly for the heterogeneity across different countries.

Data reported here, not only come from a large international registry, but also only includes patients diagnosed with BP after 2015. This differs from previously published data sets that mainly include patients diagnosed until 2016 [31, 33–35], and provides a more realistic picture of current therapy strategies in Europe. Age, sex and phenotype distribution were in accordance with available literature data [31, 35]. For those patients diagnosed in CP, median time to development of BP was 10 months shorter than what has been reported by the MD Anderson group [31]. A possible reason for this finding could be the above-mentioned different recruitment period. It could be speculated that better knowledge of the biology of CML and a wider availability of TKI in recent years prompted to quicker changes in therapy in patients with suboptimal response. This may have resulted in the selection of a higher risk population in our cohort, that progressed faster into CML-BP. An additional difference is that Jain et al. included patients with CML-BP defined according to the ELN criteria (30% blasts) [24, 29], whilst this registry included patients with CML-BP according to the WHO definition (≥20% blasts) [25]. In patients included in this registry, outcomes were dismal for both groups, strengthening the notion that CML-BP should be defined when the percentage of blasts is equal or greater than 20%.

Another finding of interest is the high percentage of patients with a de novo BP. The reasons for this can only be speculated; data present here could simply indicate a shift in proportion of patients presenting with de novo BP and BP evolving from a previous CP rather than an increase in de novo BP, as, due to higher effective therapy, less patients are progressing to BP. Further data from independent cohorts are needed to confirm this finding and corroborate this speculation. The proportion of patients presenting with MyBP and LyBP was comparable between de novo BP and secondary BP, suggesting the absence of Ph + acute lymphoblastic leukemia patients in the registry. There was a slight female predominance in patients with de novo BP as well as a higher proportion of atypical *BCR::ABL1* transcripts and a lower proportion of high-risk ACAs, suggesting a different biology behind these two entities. The presence of mutations in gene others than *BCR::ABL1* has been reported by several authors [36, 37], and has been linked both to disease evolution as well as to lower responses to TKI in the CP of the disease [38, 39]. In this multinational registry, molecular data on genes other than *BCR::ABL1* were determined only for a minority of patients, preventing at present an analysis of their impact in CML-BP outcomes. Considering the current effort within the Harmony consortium [40], collecting and evaluating the role of additional mutations in patients with CML, these data will become highly important in the future. Additionally, the availability of specific inhibitors against some of the reported mutations (e.g., FLT3, NPM1) will add additional treatment options for patients with CML-BP. It is therefore important that a complete molecular profile is performed in all patients with CML-BP.

Treatment of BP was heterogeneous. Data clearly show that in real-life multiple patients-related and disease-related factors play a role in treatment choice. Age, previous disease history, previous treatment history and the phenotype of the BP were the strongest factors that influenced treatment decision. The combination of TKI and chemotherapy was the most widely applied treatment strategy in first line, although there was an extreme variability in the type of chemotherapy associated to TKI treatment. Recently, Copland et al. showed that the combination of ponatinib and FLAG-IDA can be a safe and effective option for patients diagnosed with lymphoid or myeloid BP [41]. Other authors have reported on the efficacy of the combination of TKIs with HyperCVAD [34] or the classical “3 + 7” [42] in lymphoid and myeloid BP, respectively. In our registry there was not a preferred chemotherapy combination, and treatment was mainly decided according to patients- and disease-characteristics. The most frequently used TKI in BP evolving from a previous CML-CP were dasatinib and ponatinib, confirming the role of second and third generation TKI in BP. Imatinib was widely used in de novo BP, likely due to its favorable safety profile and restriction of TKI availability in some of the participating countries.

For those patients not proceeding to alloSCT, responses are reported to be short-term [34, 41]. In patients included in this registry, alloSCT increased the rate of high-quality responses (MR^4^ or better). As alloSCT was used in different phases of the disease and was not part of first line treatment in all alloSCT eligible patients, a clear dissection of the role of alloSCT in prolonging survival was not possible. Additional data are being collected to be able to answer this question with a longer follow up.

In the registry, 21 and 33 patients died within 3 and 6 months from BP diagnosis, respectively. This stresses the importance of an intensive supportive therapy and, for those patients that are eligible, of timely transplantation, as, differently from acute leukemias, recovery of normal hematopoiesis cannot be expected in CML-BP.

Survival in this population was dismal, with a median OS of slightly less than 2 years. Looking at survival in different subgroups data confirmed that CML-BP with a lymphoid morphology and de novo BP have better outcomes than myeloid CML-BP and BP evolving from a previous CP [31, 33, 42]. Two interesting observations were made in this cohort. Firstly, known high-risk features, such as CNS involvement and extramedullary disease, did not seem to have an impact on overall survival. Sixty-seven patients (29%) received CNS effective therapy, however, whether this finding was due to the application of intrathecal chemotherapy or to better protocols in recent years could not be unraveled. It is however an interesting suggestion, that should be further explored in larger cohorts. The second interesting observation was that the ELTS score remained predictive of an inferior survival even during BP. Patients with an intermediate or high ELTS score at the time of CML diagnosis had an inferior survival from the development of BP than patients with a low ELTS score or with a de novo BP. Thus, these patients might profit from a more intensive therapy regime and possibly from a maintenance therapy with TKI. The significance of these observations, as well as a more detailed look into possible prognostic factors, needs to be further assessed when a longer follow up will be available.

This presentation of registry data has several limitations. First, this is an observational analysis, as the registry was not powered to assess for statistically significant differences. Thus, all the data are to be intended as observational data that need to be confirmed in further analyses and on additional patient populations. Due to the nature of the registry, national differences in drug availability, as well as access to medical care and lifestyle of the patients could have impacted the results. For this reason, it is not possible, based on this data, to give general recommendations on which TKI should be preferentially used in BP. However, it is clear from the data that the choice of TKI in BP evolving from a previous CML-CP has to be based on previous therapies with a strong preference for second or third generation TKI. On the other hand, the variability of the registry gives us a realistic picture of treatment strategies. To include as many patients as possible and to reduce the risk of selecting for good performing patients in those centers activated at a later time point, also deceased patients could be included retrospectively. This could have caused a selection for “poor performing patients”, reducing the observed OS. Thirty percent of the patients included in the registry fall into this category and were mainly patients included between 2015 and 2017. However, looking at the survival of these patients compared to the one of patients included after 2018, there were no significant differences (data not shown), so that the effect of this possible bias on OS survival can be considered as negligible.

In summary, data demonstrate the heterogeneous biology and clinical presentation of CML-BP, where a standardization of treatment might not be possible. Although the possibility to apply the same treatment protocol to CML-BP patients has been advocated [41], these data suggest that treatment choice should be guided by disease- and patient-characteristics, with the aim of blast eradication. The data available did not allow at present to fully unravel the role of allogeneic stem cell transplantation. Future analyses are planned to better dissect the impact of standard dose induction chemotherapy and allogeneic stem cell transplantation in CML-BP patients. The goal is be to achieve a high quality molecular response (molecular undetectable leukemia), as this seems to be even of more value in patients with CML-BP compared to patients with CML-CP [33]. Known prognostic factors like CNS involvement have to be re-evaluated and until a score for BP is developed, the use of the ELTS score should be promoted. These findings should set the basis for further research, to improve the prognosis of this still challenging patients collective.

### Supplementary information

Supplementary tables

## Data Availability

Data are available from the authors upon reasonable request.
