# Supplementary material for: Management and outcome of patients with chronic myeloid leukemia in blast phase in the tyrosine kinase inhibitor era – analysis of the European LeukemiaNet Blast Phase Registry
Source: Leukemia. 2024 Mar 28;38(5):1072–80. doi: 10.1038/s41375-024-02204-y (PMC11073984; doi:10.1038/s41375-024-02204-y)
Supplement: Supplementary file 1 — Supplementary tables [file 41375_2024_2204_MOESM1_ESM.pdf]

Supplementary Table 1. TKI availability outside clinical trials in the participating countries

Country	Chronic phase	Blast phase
Armenia	From first line: Imatinib	First line: Imatinib
	From second line: Nilotinib	Second line: Nilotinib
	From third line: Ponatinib	Third line: Ponatinib
	Not available: Dasatinib, Bosutinib, Asciminib	Not available: Dasatinib, Bosutinib, Asciminib
Austria	From first: Imatinib, Dasatinib, Nilotinib, Bosutinib	First line: Imatinib, Dasatinib, Nilotinib, Bosutinib
	Resistant or intolerant to Dasatinib or Nilotinib: Ponatinib	Resistant or intolerant to Dasatinib or Nilotinib: Ponatinib
	T315I any line: Ponatinib	T315I: Ponatinib
	After two TKI: Asciminib	After two TKI: Asciminib
Czech Republic	From first line: Imatinib, Dasatinib, Nilotinib	All lines: Imatinib, Dasatinib, Nilotinib, Bosutinib, Ponatinib
	From second line and after special permission for health insurance company: Bosutinib	
	From third line: Ponatinib	
	T315I any line: Ponatinib	
	From third line and after special permission for health insurance company: Asciminib	
France	From first line: Imatinib, Nilotinib, Bosutinib	All lines: Imatinib, Dasatinib, Nilotinib, Bosutinib, Ponatinib
	From second line: Dasatinib	
	From third line: Ponatinib, Asciminib (not for T315I)	
	T315I any line: Ponatinib	
Germany	From first line: Imatinib, Dasatinib, Nilotinib, Bosutinib	No previous therapy with TKI: Imatinib, Dasatinib, Bosutinib
	From third line: Ponatinib, Asciminib	Previous therapy with TKI: Imatinib, Dasatinib, Nilotinib, Bosutinib, Ponatinib
	T315I any line: Ponatinib	

Italy	From first line: Imatinib, Dasatinib, Nilotinib, Bosutinib	From first line: Imatinib
	Patients resistant/intolerant to Dasatinib and/or Nilotinib: Ponatinib	Patients resistant/intolerant to Imatinib:Dasatinib
	From third line: Asciminib	Patients resistant/intolerant to at least one TKI: Bosutinib
	T315I any line: Ponatinib	Patients resistant/intolerant to Dasatinib and/or Nilotinib: Ponatinib
		T315I any line: Ponatinib
Poland	From first line: Imatinib	Any line: Imatinib, Dasatinib, Bosutinib, Ponatinib
	From second line: Dasatinib, Nilotinib, Bosutinib	
	From third line: Ponatinib,	
Russian Federation	From first line: Imatinib	Any line: Imatinib, Dasatinib
	From second line: Dasatinib, Nilotinib, Bosutinib	
	Managed access program: Asciminib	
Slovenia	From first line: Imatinib, Dasatinib, Nilotinib	From first line: Imatinib, Dasatinib, Nilotinib
	From second line: Bosutinib, Ponatinib	From second line: Bosutinib, Ponatinib
Spain	From first line: Imatinib, Dasatinib, Nilotinib, Bosutinib	No previous therapy with TKI: Imatinib, Dasatinib, Bosutinib
	From third line: Ponatinib, Asciminib	Previous therapy with TKI: Imatinib, Dasatinib, Nilotinib, Bosutinib, Ponatinib
	T315I any line: Ponatinib	T315I any line: Ponatinib
Sweden	From first line: Imatinib, Dasatinib, Nilotinib, Bosutinib	No previous therapy with TKI: Imatinib, Dasatinib, Bosutinib
	From third line: Ponatinib, Asciminib	Previous therapy with TKI: Imatinib, Dasatinib, Nilotinib, Bosutinib, Ponatinib
	T315I any line: Ponatinib	T315I any line: Ponatinib

* from first line refers only to patients diagnosed with de novo blast phase. Patients whose blast phase is an evolution of a previous chronic phase are automatically considered from second line (according to the number of chronic phase therapies received).

Supplementary Table 2. Characteristics of patients with atypical *BCR::ABL1* transcript

	BCR::ABL1	**Age at onset of BP (years)**	**Phase CML at diagnosis (CP vs BP)**	**Morphology /Phenotype**	***BCR::ABL1*** **Mutations**	**CNS/EM involvement**
1	**e1a2**	40	BP	Lymphoid	No	No
2	**e1a2**	44	BP	Lymphoid	No	Yes
3	**e1a2**	57	BP	Lymphoid	No	Yes
4	**e1a2**	67	CP	Myeloid	Yes	No
5	**e1a2**	62	CP	Lymphoid	No	No
6	**e1a2**	48	BP	Myeloid	No	No
7	**e1a2**	26	BP	Myeloid	No	No
8	**e6a2**	74	BP	Myeloid	No	Unknown
9	**e8a2**	76	CP	Unknown	No	No
10	**e19a2**	53	BP	Myeloid	No	Yes
11	**e19a2**	54	BP	Myeloid	No	No
12	**e13a3**	60	BP	Lymphoid	No	No

CML: chronic myeloid leukemia; CP: chronic phase; BP: blast phase; Y: yes; N: no; CNS: central nervous system; EM: extramedullary manifestation(s)

Supplementary Table 3. Type of treatment administered for first treatment of CML-BP

**Treatment combinations**	**N (=232)**	**N (=145) (secondary BP)**	**N (=87) (*de novo* BP)**
TKI+Ctx+alloSCT	35 (15.1%)	23 (15.9%)	12 (13.8%)
TKI+alloSCT	10 (0.4%)	7 (4.8%)	3 (3.4%)
TKI+Ctx	99 (42.7%)	55 (37.9%)	44 (50.6%)
TKI alone	49 (21.1%)	31 (21.4%)	18 (20.7%)
TKI+HMA	0	0	0
TKI+HMA+alloSCT	0	0	0
TKI+HMA+alloSCT+Ctx	0	0	0
Ctx alone	32 (13.8%)	22 (15.2%)	10 (11.5%)
Ctx+alloSCT	2 (0.9%)	2 (1.4%)	0
alloSCT alone	3 (1.3%)	3 (2.1%)	0
Best supportive care	2 (0.9%)	2 (1.4%)	0
**Type of TKI**	**N**		
Dasatinib	75	52	23
Ponatinib	31	31	0
Imatinib	62	13	49
Nilotinib	19	15	4
Bosutinib	4	3	1
Others*	2	2	0

CML-BP: chronic myeloid leukemia blast phase; TKI: tyrosine kinase inhibitor; Ctx: chemotherapy; alloSCT: allogeneic stem cell transplantation; HMA: hypomethylating agents

*Others: midostaurin (n = 1), unknown (n=1)

Supplementary Table 4. Type of treatment administered for CML-BP in all treatment lines

**Treatment combinations**	**N**	**N (secondary BP)**	**N (*de novo* BP)**
TKI+Ctx+alloSCT	109	65	44
TKI+alloSCT	12	8	4
TKI+Ctx	69	36	33
TKI alone	26	19	7
TKI+HMA	1	1	0
TKI+HMA+alloSCT	1	1	0
TKI+HMA+alloSCT+Ctx	6	6	0
Ctx alone	9	9	0
Ctx+alloSCT	3	2	1
alloSCT alone	2	2	0
Best supportive care	2	2	0
**Type of TKI**	**N**		
Dasatinib	133	76	57
Ponatinib	95	69	26
Imatinib	80	19	61
Nilotinib	46	30	16
Bosutinib	19	13	6
Others*	16	13	3

CML-BP: chronic myeloid leukemia blast phase; TKI: tyrosine kinase inhibitor; Ctx: chemotherapy; alloSCT: allogeneic stem cell transplantation; HMA: hypomethylating agents

*Others: asciminib (n = 14), PF-114 (n = 1), midostaurin (n = 1)
